# Cultural Practices of Mothers and Babies during the Postnatal Period: A Qualitative Study in Rural Bangladesh

**DOI:** 10.3390/ijerph21101344

**Published:** 2024-10-11

**Authors:** Nusrat Jahan, Md Shahidul Islam

**Affiliations:** School of Health, Faculty of Medicine and Health, University of New England, Armidale, NSW 2351, Australia; njahan3@une.edu.au

**Keywords:** cultural practices, mothers, babies, postnatal care, postnatal period, Bangladesh

## Abstract

This study describes the experiences of women in rural Bangladesh, and the cultural practices related to how they take care of themselves and their babies, in the early postnatal period. Data were gathered through immersion in the field for six months, participant observation, document collection and in-depth interview techniques to gain a deep understanding of women’s everyday lives and practices during the postnatal period. In-depth interviews were undertaken with 28 mothers who had had at least one live child within the five years before the date of data collection, in *Purba Sharifabad* village of the Barisal Division in Bangladesh. The key findings of this study highlight the role of culture in the experiences of women during the postnatal period, and reveal that cultural practices, beliefs and traditions are deeply embedded in the lives of rural women. This study focuses on cultural practices which have an effect on the choices of women regarding care and support from healthcare facilities. Culture, together with poverty and low levels of education, leads them to resist healthcare from a skilled birth attendant and encourages them to believe in and trust traditional care practices during the postnatal period. Therefore, the findings of this study may contribute to future policies, planning, programs and research in Bangladesh by providing an understanding of the importance of culturally and socially rooted traditional beliefs and cultural practices. These need to be addressed for maternal and newborn healthcare initiatives to be effective, particularly in rural Bangladesh.

## 1. Introduction

Childbirth and the immediate postnatal period represent a major transition in a woman’s life in all societies, and is socially marked and culturally constructed. Childbirth is not only a physiological phenomenon; it incorporates the social and cultural values of the society and people involved. The practice of cultural rituals, traditions and ceremonies after childbirth are very common throughout the world. Childbearing poses inherent risks, especially for women who live in poverty and who have poor access to skilled birth attendants (SBAs) and quality healthcare. According to the World Health Organization, many thousands of women die every year from preventable causes related to childbirth. By far the majority of these deaths (95%) occur in developing countries, especially in rural areas and among poorer communities [1]. 

Bangladesh, as a developing country, became a signatory to the WHO Safe Motherhood strategy in 1988, the Millennium Development Goals (MDGs) in 2000 and the Sustainable Development Goals (SDGs) in 2015 in an attempt to reduce maternal and infant mortality rates and improve maternal and newborn health. Despite improvements in some sectors, maternal and infant mortality rates are still high, especially in rural areas. For example, while Bangladesh aimed to achieve a reduction in the maternal mortality ratio by three-quarters between 1990 and 2020, evidence suggests that despite progress (from 569 in 1990 to 165 in 2019 per 100,000 live births), the maternal mortality ratio lagged behind in rural areas compared to in urban areas by 68 deaths per 100,000 live births [2]. Most mothers in Bangladesh do not receive postnatal care services from SBAs, either for themselves or their babies, despite the implementation of safe motherhood strategies and the implementation of the MDGs and SDGs. Safe motherhood advocates that the use of SBAs has been important in improving the outcomes of women and their babies during childbirth and the postnatal period. According to the Bangladesh Demographic and Health Survey 2022, the care of an SBA and hygienic conditions during childbirth are also important in reducing the risk of physical difficulties for both mothers and babies [3]. While it was important to increase the number of births taking place in safe and hygienic environments and with the care of SBAs, the number of childbirths occurring with such facilities is still low in Bangladesh. For example, the Multiple Indicator Cluster Survey 2019 states that around half of all mothers received postnatal care from SBAs or had access to quality healthcare services when difficulties arose [2]. 

Only 55% of mothers and 56% of infants received postnatal care from a medically trained provider within two days of delivery. Likewise, only 13% of mothers with noninstitutional deliveries received postnatal care from a medically trained provider within the first two days after childbirth. The data show that the rate of postnatal check-ups of mothers from a medically trained provider within the first two days after birth increased from 53% in 2017 to 55% in 2022. Similarly, the rate of children’s postnatal check-ups within the first two days increased from 53% in 2017 to 56% in 2022. Similarly, the neonatal mortality rate is still high in Bangladesh. The neonatal mortality rate in Bangladesh only reduced from 27 per 1000 live births in 2017 to 20 in 2022 [3]. 

The reasons for the poor utilization of postnatal care services in Bangladesh have been raised by several studies. For example, Aziz et al. indicated that there are many cultural and economic barriers that delay Bangladeshi women from accessing early postnatal care services [4]. In another study, Akter et al. also indicated that religious and traditional cultural beliefs and practices are the major obstacle to improving postnatal care services for women in Bangladesh [5]. Studies conducted in rural Bangladesh shows that social processes and cultural beliefs and practices play a prominent role in affecting a mother’s experience and shaping mothering behavior [6,7,8]. As Bangladesh is a patriarchal Muslim society, various social and cultural norms play a profound role in childbirth and the postnatal periods [9,10,11,12]. Yet, most maternal and newborn health research on this important time in a woman’s life has focused on the physiological processes associated with childbearing. This study uses a qualitative approach to uncover the experiences of women, and the cultural practices related to how they take care of themselves and their babies in the early postnatal period in rural Bangladesh. This study focuses on culture as a major concern in understanding women’s experiences of childbirth and postnatal care. In rural Bangladesh, studies of cultural practices in postnatal care appear to have stayed at the level of identifying rather than describing practices. This study enabled us to describe early postnatal care experiences of mothers in rural Bangladesh, which may help develop further policies, strategies and programs to improve maternal and newborn health in rural Bangladesh.

## 2. Methods

An ethnographic method was used for this study. Data were gathered through immersion in the field for six months (June–November 2011), participant observation, document collection and in-depth interview techniques to gain a deep understanding of women’s everyday lives and practices during the postnatal period. In-depth interviews were undertaken with 28 mothers who had had at least one live child within the five years before the date of data collection, in *Purba Sharifabad* village in the Barisal District in Bangladesh. 

### 2.1. Entering the Field, Observing Participants and Their Activities

The first author communicated with a nutrition worker at the Voluntary Organization for Social Development (VOSD) who assisted her as a gatekeeper in getting access to the participants. She collected documents related to mothers and babies born within the last five years from her registered book, including names and addresses. The first few days after meeting with the gatekeeper, she roamed the village to observe people’s everyday activities. She also visited women and their families in their houses to introduce herself at the addresses she had found in the nutrition worker’s (gatekeeper’s) book. She also took the gatekeeper with her because her familiarity played a great role in making relations easy between her and the rural women. After being introduced to the women, she told them about the research. She also assured them that she would not disrupt their normal activities, but would observe and interact with them. After knowing her intentions, most women were enthusiastic about her work and asked questions about her work. She maintained good relations with them by acting in an intimate way. She tried to become close to them so that they considered her an insider to their culture. She did this so she could better observe the social lives of these women, participate in day-to-day activities and actions and elicit the mothers’ understandings and experiences.

During the field work, the first author planned to observe what women did during the early postnatal period. After hearing the news of a baby’s birth one morning, she quickly went to the house. She saw that both mother and baby were lying on the jute mat on the floor, covered by a cotton quilt and *katha* (a thick bed cover made of patched cotton cloth). She could not directly enter that room as they were in seclusion. First, she had to warm her hands on the fire, which was lit in that room. After entering the room, she saw that the baby was sleeping and a bundle of items were wrapped with a piece of cloth kept near the baby’s head. After greeting the mother and asking how she was feeling, the first author asked what was inside the bundle. She saw a small piece of iron, broomsticks and a piece of garlic. She was curious about the other things in the room. She noticed that some pieces of cow bone and a small matchbox were kept near the baby’s head, out of fear of being attacked by an evil spirit. She observed a round black mark on the baby’s forehead, so that no evil eye could harm the baby. 

### 2.2. Rapport Building

To establish a good rapport with the villagers, the first author always tried to be part of that community. She could easily communicate with the participants in their local language, thus enabling her to better understand their situations and build rapport. She also tried her best to pronounce words in the local dialect, and to explain things in the native language. When she visited the participants’ houses, she always talked to the senior members of the household first, such as the mother-in-law and father-in-law. She informally talked with them about a variety issues, including health, food and their family. Over time, she chatted with them for hours so that she could gain access to the participants. As she also spent considerable time with the participants, her relationship was easy and relaxed. 

### 2.3. Selection of the Participants and Interview

From the VOSD’s official record, the first author found a total of 54 women who had given birth within the last five years. As a first step, 35 women who met the inclusion criteria were identified; the other 19 were excluded for reasons such as migration to another area, traveling, becoming pregnant or being below the age of 18. During the participant observation stage of the study, the first author initially communicated with 35 women and 3 women rejected her invitation. She maintained good relations with the 32 women who showed interest in joining the study. After a number of visits, she offered them the opportunity to participate in an in-depth interview. She stressed to all participants that their participation in this research was voluntary and depended on their willingness. Ultimately, 28 women agreed to participate in in-depth interviews. 

### 2.4. Experience during Interview

When women gave their consent to be interviewed, the first author organized the time, date and place according to their wishes. Afterwards, she visited their home and again verbally shared detailed information to clarify her main intention and the research objectives. She also gave an information sheet and consent form to read and sign. Most interviews took place at the participant’s house, which was suitable for them. Most participants chose their own room to talk to the first author about childbirth. In some cases, she conducted the interview sitting on their bed. Sometimes she sat on the *piri* or *madur* (mat) alongside the participants. 

During interviews, she was very careful to help participants feel relaxed and comfortable. Although she developed friendly relationships with the participants, she started the conversation with broad topics like how the mother’s day was, the baby and health, as doing so encouraged natural discussion and made participants feel easy. She did not rush into the main discussion. She spoke in a pleasant tone of voice so that they felt comfortable describing their experiences. She also responded to their emotions, such as consoling them when they cried, and incorporated humor when needed.

In an attempt to understand the mother’s experience of childbirth and early postnatal care, she started raising some issues of childbirth following the semi-structured interview guidelines that she had prepared for in-depth informal conversations in the form of narratives. In the interview, she talked with mothers in detail about their childbirth and the first six weeks after birth. She also asked what mothers did to look after their babies and themselves in the first six weeks after birth. She gave participants a full chance to share their experiences. She never tried to influence them with her own ideas. Her role was as an active listener and an effective and efficient recorder. 

### 2.5. Ethical Considerations

Prior to conducting field work, we gained ethical approval from the Human Research Ethics Committee, University of New England, Australia. The information sheet for participants was translated into the Bangla language so participants could understand the details of the research. For participants who could not read the information sheet and consent form, the first author verbally explained the purpose and procedures. She also took their written consent paper with their signature. She informed them that personal information gathered during the interview would remain confidential. She also informed them that all identifying information (including audiotapes and transcripts) would be kept in a locked place for privacy and confidentiality, and that their names would be replaced by a number so that they could not be identified.

### 2.6. Dealing with Transcripts and Analyzing the Data

During field work, the first author transcribed the audio files and translated them from Bangla to English. She read the transcripts carefully, line-by-line, and underlined the key words to identify relevant codes for this research. She also coded participants’ statements by using a word, phrase or clause found in the text, and then placed the codes either to the right or left of the transcripts. She used colored markers to highlight similar codes. Thus, after obtaining a clear idea about promising codes from the transcripts, she used a systematic qualitative data management and analysis program, NVivo 9, for coding the data. An interlinked relational network was then developed in logical combinations as a tool for the identification of common themes. Meanings were formulated to produce clusters of themes. Themes were compared within and across categories to establish consistency. 

### 2.7. Demographics of the Participants

All participants were married, except for one who lived separately from her husband, and another who was a widow. The age range of participants was 18–35 years. The average age was 25.79 years. In this research, the level of education of participants varied from no education to year 12, with an average of 6.96 years’ education. Most women did not go to secondary school. All had left school because of their marriage or to pursue household work and maintain the family. The number of children that participants had given birth to varied from one to five. The average number of children was two, with twenty girls and eight boys. The age of the children varied from 44 days to 5 years, with an average of 1.5 years.

All participants were Muslim as the study was conducted in a village where Islam is the dominant religion. All participants were housewives except one, who was employed in an NGO as a field worker, and earned BDT 2000 monthly (USD 17). Therefore, all participants depended on their husband’s income and earnings. Two participants did not mention their husband’s income, as one of the participant’s husbands had died, and another one was separated and lived with her parents. The level of income of the participants’ husbands (*n* = 26) varied from BDT 1200 (USD 10) per month to BDT 20,000 (USD 170) per month, with an average of taka BDT 6871.42 (USD 58). The economic status of the participants in this research was considered to be the average income of the husband. Five participants informed me that they had a good income with which to maintain the family. Eight participants reported that they just managed to maintain their family. The remainder of participants (50%) stated that their income was much lower than they needed to maintain the family. They had to depend on others’ support.

Eleven (28%) women gave birth in hospital. Another 13 (46%) gave birth at their husband’s house, and 4 gave birth at their mother’s house with the assistance of a *dai*. A total of 13 (46%) participants told me that they had had a medical check-up after childbirth. Of these women, eight received postnatal care from SBAs, and the remaining five received care from the nutrition worker and community health worker.

## 3. Results

### 3.1. Food Restrictions

In this study, food restrictions or taboos were visible in the postnatal period. Almost all participants (85.7%) revealed that they maintained food restrictions during the postnatal period, which deprived them of a proper diet and nutritional intake. For example, most participants eliminated fish from their diets, even though fish is the primary source of protein for rural people in Bangladesh. Nineteen of the twenty-eight participants mentioned that they did not eat fish for at least seven days after childbirth because they believed it would be harmful to them and their babies. Participant 10 said, ‘My mother-in-law strictly prohibited me from eating fish after childbirth, as having it would be harmful to the baby’. 

Participants were restricted from eating other nutritious foods, including some vegetables, meat (especially beef), bananas, milk and fruit. For example, some leafy vegetables like spinach were not allowed to be eaten after childbirth because they caused stomach ache. Participant 14 said:

When I came home from the hospital after giving birth, my mother-in-law forbade me from eating spinach and fried green chili because eating these foods could be a cause of bellyache for the baby.

After childbirth, many participants mentioned that they did not drink milk. Participant 12 said, ‘I did not drink milk because it delays the drying of the baby’s umbilical cord. My mother forbade me to drink milk’. Taboos on eating beef were also seen, out of fear of stomach ache, allergies and decreasing breast milk supply. 

### 3.2. Food after Childbirth

Participants were in seclusion immediately after birth, and several dietary restrictions were imposed on them. The participants said that the most common foods after childbirth were mashed foods like potato, pawpaw, black cumin and green banana. Most participants (24 out of 28) said they ate mainly mashed foods for at least one week after seclusion, as these reduced pains and helped heal the birth passage. For example, participant 2 said:

After giving birth, I ate several mashed foods, including *kalijira vorta*, mashed mustard seeds, mashed potato and mashed green banana, because these mashed foods helped reduce body pain and heal the birth passage.

Some participants also stated that they ate *kalijira vorta* not only to reduce body pain but also to increase breast milk. For example, participant 7 said, ‘I ate *kalijira vorta* to reduce my body pain and to increase my breast milk’. 

### 3.3. Hygiene Practices after Childbirth

Most participants maintained cleanliness after giving birth, although some followed unhygienic practices, such as giving birth in a dirty place and using a used razor blade during home births. Just after childbirth, most participants used washed cloth rags for postpartum bleeding because they were unable to buy expensive sanitary pads. A few economically well-off participants used sanitary pads. As participant 24 said, ‘I used sanitary pads for my postpartum bleeding. The doctor advised me to use sanitary pads because used rags never get properly clean’. Usually, participants used the same piece of cloth repeatedly for postpartum bleeding. After every use, they washed them and dried them in a hidden dark place, so that no man would see. Participant 16 said, ‘After childbirth I used clean cloths to absorb dirty blood. Every day I cleaned the rags with soap and then dried them in a dark hidden place so that no man could see the dirty rags’. 

### 3.4. Protection from Evil Spirits

Women in this village were afraid about evil spirits during pregnancy, childbirth and the postnatal period, as they seemed more vulnerable to attacks from evil spirits than other people. Usually, people treat *jin* and *pori* as evil spirits. *Bhoot* are also considered evil spirits. Rural people believed that these creatures could harm human beings. Participant 24 said:

My mother told me that *jin and pori* were real, as their names are written in the Quran. That is why my mother gave me a *tabeez* as a bodyguard for my baby’s throat, so that *jin* and *pori* could not harm my baby.

Participants performed several rituals for protection from evil spirits, including using *tabeez* (amulets containing holy words or a spell to protect against evil spirits)*,* lighting a fire, exorcizing, restricting when they went outside, keeping something near the baby’s head when going outside (such as a cow’s head, a broomstick or a piece of iron). All participants mentioned that they did these things to protect their babies from the harmful effects of evil spirits.

### 3.5. Using Amulets

Mothers and babies wore *tabeez* as protection against *jin* and *pori*. They believed that *tabeez* were the most powerful protection against evil spirits. In this research, 26 participants talked about using *tabeez* either for themselves or their baby. Usually, women collected *tabeez* from a famous spiritual person, such as a *fakir* or *huzur*. Village women believed that *tabeez* can protect not only from evil spirits but also from other problems, such as disease and the evil eye. Some participants mentioned another type of *tabeez* used for a newborn baby. After the dried umbilical cord had fallen off the baby, it was kept in a *tabeez* as protection from evil spirits. Elders in extended families instructed women to use these *tabeez,* as they believed that the baby’s umbilical cord had a direct connection to the mother. They believed the umbilical cord was very sensitive to evil spirits if it was just discarded. Therefore, they believed it should be kept in a hiding place, like a *tabeez,* which also protected the baby from *jin* and *pori*. Participant 20 said:

After cutting the cord, the *dai* tied the rest of the umbilical cord with thread. Within three days, my baby’s umbilical cord was dried. Then it fell off. My mother-in-law told me to keep that dried cord hiding in a *tabeez* for 18 months, as it could be easily affected by *jin* and *pori* due to its direct bond with the mother’s womb. I followed their instructions. As a result, my baby was protected from *jin* and *pori.* After 18 months I threw that *tabeez* into the water, because it was also the rule of this *tabeez*.

### 3.6. Keeping Something near the Baby’s Head

After childbirth, mothers in the village follow the rituals of seclusion. During this period, mothers follow several practices as they are believed to be more vulnerable from the harmful effects of evil spirits. Keeping something near a baby’s head—such as cow’s teeth, a piece of garlic, a piece of iron and a match stick—is a common practice in this village against evil spirits. In this research, 26 participants described that they kept various things near their baby’s head. Among them, the most common were cow’s teeth, a small piece of iron, a match stick box and a piece of garlic. A few participants mentioned keeping a broomstick beside the baby as a protector against evil spirits. People believed that evil spirits became frightened when these kinds of materials were kept near a baby. For example, participant 27 said:

I kept the match stick and broom stick in the matchbox near my baby’s head so that the baby could be safe from *bhoot*. My mother collected all these things. I also kept an exercise book and a pen in the bed so that the baby would become educated in future. On the roof of my house I kept cow’s teeth and iron. I always kept a small piece iron with my baby. When I went outside, the iron protected me from *bhoot* because *bhoot* get frightened to see the metal.

### 3.7. Restrictions on Going outside at Particular Times

In this study, women could not go outside the house at some specific times in the postnatal period, specifically during the seclusion period. Villagers called these restricted times *khan*. According to the participants, they were strongly constrained from going out during *khan,* which were at dawn, noon, in the evening and at night. Going outside at these times was restricted because *jin* and *pori* frequently moved around at these times. It was believed that *jin* and *pori* would do harm to babies and mothers if they found them outside. For example, participant 6 said, ‘I did not go outside all the time. My aunt-in-law forbade me from going outside at dawn, in the evening and at night. She also forbade going outside at 12 pm and 3 pm because *jin* and *pori* normally moved at that time’. 

In this village, there is a belief that *jin* and *pori* are frightened to see fire and cannot come close to it, as they are made of fire. Therefore, rural mothers lit a fire in the baby’s room so that no evil spirit could do harm to their babies. This practice continued during seclusion. According to the participants’ descriptions, the fire was kept in the room where the mother and baby slept together. So, before entering the room, everyone had to heat their hands slightly, as they believed that this practice could protect the baby from harm. For example, participant 5 said, ‘Many people came to see my baby after his birth. I kept a burning *hurricane* (lantern) in my room because it is a belief that *jin* and *pori* cannot harm the baby if people enter the room after warming their hands on fire’. 

More than half of the participants lit fires to protect against evil spirits. However, some participants did not like to light a fire in the baby’s room because they thought that this might create smoke that could harm the baby’s lungs. For instance, participant 13 stated, ‘I did not light *dhup* in my room because *dhup* creates smoke, which goes directly to a baby’s brain and causes harm’.

### 3.8. Exorcism

Exorcisms were a common practice in this village for expelling evil spirits. Exorcisms happened when differences in a baby’s behavior were observed, as these were thought to be the outcome of an evil spirit attacking. To be rid of the influence of the evil spirit, village women sought support from a *fakir,* who had the knowledge of exorcism. During exorcisms, the *fakir* usually used *athali pata*, undid the mother’s hair and uttered a *mantra*. Participant 23 stated:

The *fakir* exorcised my baby four to five times by *athali pata* and uttering some *mantra,* because my baby cried a lot at night and she did not want to drink breast milk. I thought that *jin* and *pori* might attack my baby. Therefore, I went to the *fakir* for his support. During the exorcism of my baby, the *fakir* told me to undo my hair as it was one of the rules of exorcism.

### 3.9. Using Kajal

Women were not only scared about evil spirits but also were afraid of *najar* (evil eye) from other people. The participants stated that the evil eye could harm the baby. Therefore, they used *kajal* by putting a *tip* (a bold, round black spot) on the baby’s forehead. Twenty-two participants mentioned using *kajal* on their baby’s forehead. For example, participant 13 said, ‘I used *kajal* as a *tip* so that my baby could not suffer from people’s evil eyes. I also used powder on the *kajal* so that it could not be spread. My baby was very beautiful. For this reason, I took protection by using *kajal*’. 

### 3.10. Practice of Seclusion

After childbirth, women in this village generally confined themselves with their baby in a single room, locally known as *ahuj ghor*. They were treated as impure because of giving birth and postpartum bleeding. There were two reasons given for following the practice of seclusion. First, women were considered impure and polluted, so they kept themselves away from other people, and second, to protect their baby from evil spirits. Participants followed several traditional practices to prevent the ill effects of evil spirits, including using *tabeez,* lighting fires, exorcisms, using *kajal*, going outside at certain times only and keeping certain items near the baby’s head and when going outside.

Participants maintained several practices during seclusion, including the maintenance of food taboos, living separately from the family (including the husband), not touching others (including the husband), restrictions on going outside, lighting a fire in the room and not making food. The seclusion period gave women a rest, as they remained inside the house all the times out of fear of being attacked by evil spirits. After coming out of seclusion, the women started performing their household chores. It was for this reason that many participants could not follow seclusion for longer. In this study, most participants maintained confinement rules after childbirth. Participant 20 described her experiences:

As I was impure after childbirth, I was not permitted to cook food because others could not eat my cooking. I did not perform any household chores for one week. I also never touched others’ food due to my pollution. I used separate glasses, plates and jug. I ate in my bed, as my mother served food in my bed. Mostly I passed my time in bed with my baby. I rested at that time. I only went outside for a shower and to use the toilet which is not attached to our house.

It was also believed that food prepared by newly birthed mothers would be polluted and harmful, as they were treated as impure. For example, participant 11 said:

During the 40 days I never went to the kitchen to cook. It was a rule. So my husband did not permit me to go there as I had *choot* (impurity from postpartum bleeding). If I had prepared food for my husband during those days, it would have been harmful for him as I was impure.

### 3.11. Following Common Rituals for Baby after Childbirth

#### 3.11.1. Bathing

Participants mentioned that they bathed their babies immediately after birth with warm water and a silver and gold ornament. For example, participant 10 said, ‘After my baby’s birth, my mother-in-law bathed my baby. She used light warm water and dipped a silver and gold ornament into that water. She did this as it was the rule for the first shower for a baby’. For the baby’s bath, women mostly used warm water heated by sunlight. They did this because they thought this process was more natural and would not cause a cold. For example, participant 16 stated:

For my baby’s bath the *dai* kept water in the sunlight so that it could be warmed by the heat of the sun. Then she used that hot water for my baby’s bath because it was good for my baby’s sensitive body. Having a shower with this warm water also helps the baby not get cold.

#### 3.11.2. Feeding

While breastfeeding has increased nowadays, some cultural beliefs still predominated among the women in this village that led to babies being fed other things just after the birth. For example, participants followed a common practice of feeding honey, as they thought that this could help the baby to speak sweetly. Participant 23 said, ‘My mother-in-law fed honey and water mixed with sugar candy to my baby just after birth so that the baby could speak sweetly’. 

Participant 27 said that she fed goat’s milk to her baby, as this was believed to protect it from cold. She also mentioned some other traditional practices related to feeding. She said:

Just after giving birth, my mother gave mustard oil to my baby to clean the mouth. Then I tried to feed milk to my baby. As my baby did not get enough breast milk, I fed goat’s milk to my baby because if a baby drinks goat’s milk, she or he will not get cold. However, when my baby started feeding well, I did not feed anything except breast milk.

In this study, participants were not counseled adequately in both the antenatal and the postnatal period about the importance of exclusive breastfeeding. For example, participant 17 did not have enough knowledge about exclusive breastfeeding and colostrum. As her baby received only a little colostrum after birth, she was worried and started feeding formula. She said:

In the first three days after my baby’s birth, my baby did not get sufficient breast milk. My baby only got a little amount of *shal doodh* (colostrum). So I fed Lacto Zen-1 (baby formula) to my baby although the doctor forbade me to feed alternative milk. But what can I do? Baby was screaming. For this reason, I gave her formula.

A few participants did not feed colostrum to their babies. For example, participant 2 said, ‘I did not feed the first milk to my baby because my mother-in-law told me that feeding the first milk to the baby is not good for her health, as it causes fever and illness. Besides, the baby will not be able to digest that milk’. Conversely, participants who were economically well-off and educated initiated early breastfeeding just after childbirth. For example, participant 24 said, ‘I knew the significance of feeding colostrum to the baby just after giving birth from watching television and reading books. Therefore, I started breastfeeding my baby immediately after her birth’.

#### 3.11.3. Umbilical Cord Care

Most participants applied a substance to the cord immediately after cutting it. The most common substances used were boric powder and mustard oil. Eleven participants said that they used boric powder. For example, participant 20 said, ‘The *dai* told me to use boric powder to dry the cord. Before using the boric powder, I used Dettol to clean my baby’s navel’. Mustard oil was a traditional cord care practice performed by village women. Seven participants reported that they used mustard oil on the cord stump. Participant 18 said:

The *dai* cut my baby’s umbilical cord. Then she tied the rest of the cord with thread. However, my baby’s cord did not dry quickly. Then I used mustard oil on the cord. Before using the mustard oil, I kept it in an oyster shell with a small piece of garlic. Then I heated the oil on the lamp.

#### 3.11.4. Care of a Baby’s Rash

Many participants took special care of their babies when they found a *nunti* on their bodies after birth. They followed several cultural practices to cure *nunti*. The most common was spraying water on the baby’s body. This was a mixture of several materials, such as milk, *kacha holud* (raw turmeric), *durba ghash* (paddy grains), *neem pata* (Margosa plant) and water. Other practices included keeping the baby away from women who had their period. Twenty participants followed these rituals and the rest did not as their babies did not experience *nunti* within six weeks of childbirth. For example, participant 10 said:

When my baby experienced a *nunti*, I practiced some rituals to cure my baby, as suggested by my mother-in-law. First, I dipped a silver and gold ornament into water. I also mixed milk and *neem pata, kacha holud* and *dubba ghash* in that water. Then I sprayed that water on my baby’s body so that the germs would be eliminated quickly. I also hung 101 leaves of the jackfruit tree in front of my house so that the germ could not enter the house. My mother-in-law kept *neem pata* in my bed as protection against germs.

Women in this village had to follow some rules for themselves when treating their babies for *nunti*. They did not eat fish as their elders forbade it. Another restriction was around menstruating women. A woman who had her period was strictly restricted from entering a house with a baby, because it was believed that women were impure at this time, which could make the *nunti* worse. For example, participant 5 said:

When my baby had a *nunti*, I was aware of women who had their period. For this reason, I always asked women about their purity and kept my baby away from them, because those women could be harmful for my baby if they touched her or came to see my daughter, due to their pollution.

#### 3.11.5. Shaving the Hair

After childbirth, shaving the baby’s hair was one way of getting rid of impurity. A newborn baby’s hair was regarded as impure, and people believed that after shaving it, the baby would be safe from the effects of evil spirits and would be regarded as pure. For example, participant 5 said:

Seven days after childbirth, one of my neighbours shaved my baby’s hair. I gave her permission to shave the hair because it was impure. To keep the first hair for a long time is not good for a baby. *Jin* and *pori* can easily attack a baby who keeps their first hair for many days.

## 4. Discussion

This study is concerned with the cultural beliefs, rituals, practices and prohibitions that persisted among mothers and babies during the postnatal period. The findings indicate that most women followed food restrictions after childbirth. In this study, culture had the most significant effect on women’s behavior and practices related to postnatal care, and it plays a crucial role in the construction of habit because culture is embodied in its forms such as the way people interact, communicate and practice their customs, beliefs and traditions. For example, most women followed food restrictions because of their traditional beliefs about food consumption during the early postnatal period. These are often restrictive and negatively affect mothers’ and babies’ nutrition. The food taboos identified in this study are consistent with the findings of other previous studies [6,7,8,9]. 

During the postnatal period, most women avoided many nutritious foods, such as a variety of fish, meat, certain vegetables, milk, chili and sour fruits. This was done for specific reasons for each food, and was guided and influenced by the practice of elder female relatives. These women disseminate their knowledge through their experiential knowledge and their past experiences which they acquired from their family through observations and suggestions from their seniors. They believed that eating certain foods had a bad effect on the bodies of mothers and babies. For various cultural reasons which play an important role in the formation of habit, women’s opportunities to eat nutritionally rich foods were limited. For example, many women did not eat fish during the early postnatal period because of their internalized dispositions of traditional beliefs that they might become vulnerable to *jin* and *pori* if they ate fish. In addition, mashed foods like potato, pawpaw, black cumin and green banana were the most common foods that women ate during the postnatal period. Decisions regarding food restrictions were made by older female relatives, who played a crucial role in providing the women with knowledge and care. These findings are consistent with earlier studies from Bangladesh [4,5,6,7,8,9].

Food taboos were commonly followed by women in this study. However, some had more education and therefore more capacity to make decisions based on exposure and engagement with wider sources of health information. In such cases, a woman’s education played a significant role in changing their behavior and their belief that they were not obliged to follow their elders’ advice.

This study found that most women kept good hygiene, keeping their bodies, houses and surroundings clean, bathing with hot water using antiseptic liquid and using sanitary napkins. While a few women mentioned using a sanitary napkin for postpartum bleeding, most wore a dirty piece of rag because of a lack of money. These rags were generally re-used without washing them with soap, and kept in a dark hidden place so that no-one could see them. This put women at risk of infection. Rural women’s poverty was a barrier to maintaining hygienic practices and their shame was evident in the act of hiding their rags. Ijdi et al. describe how it was really tough for women to follow the hygiene rules recommended by SBAs [13].

This study found that the postnatal period is characterized by several cultural beliefs and practices that are commonly marked by seclusion. After childbirth, the traditional practice of the seclusion of both women and babies was followed to protect them from the danger of evil spirits. They confined themselves in a single room from 7 to 40 days, for two significant reasons. First, the women believed that during the seclusion period, they and their babies were more vulnerable to the harm of evil spirits. Second, they were both regarded as impure and polluted. As women were believed to be vulnerable to evil spirits [4,5,14,15], to protect them, many rituals and practices were followed, including using amulets, lighting a fire, exorcism, restrictions on going outside and keeping something near the baby’s head and when going outside. Some other studies had also found the same [6,7,8,11,16,17,18]. Additionally, as childbirth is considered a period of pollution, women and their babies in this village were observed to be linked to ideas of purity and pollution during the postnatal period [10,11,13,14,18,19]. Therefore, the findings also revealed that mothers and babies became pure after completing the seclusion period through shaving the baby’s hair and cleansing the body. Aziz also showed similar results in his research [4].

The role of culture was also intrinsically seen in postnatal care practice for babies after childbirth. Women in this study followed where the embodied dispositions of women reflected deeply on their lives and the way they believed others saw them. According to Bidet, ‘… [T]he culture (… any group) as it is internalised by the individual in the form of durable dispositions that are at the basis of his/her behaviour’ (p. 120) [20]. Women practiced some common rituals guided by elders, including bathing, feeding, shaving their baby’s hair, caring for their umbilical cords and protecting themselves from cold. For example, a harmful cord-cutting practice was followed, where instruments such as contaminated razor blades are used, because some women are still unaware of the importance of using hygienic equipment to prevent infections. The most common substances used on the cord were turmeric and boric powder. These findings are consistent with previous research [21,22,23]. 

Breastfeeding is recommended for improving babies’ nutrition and development, and to help build immunity from common infections. According to the WHO, to ensure safe motherhood, mothers are recommended to initiate breastfeeding within one hour of childbirth and to continue exclusively for the first six months of life [1]. The early initiation of breastfeeding is key to a baby’s survival [23]. Despite the nutritional value of breast milk, it has been observed that some mothers delay the practice of breastfeeding after childbirth. In this research, mothers mentioned some barriers to early breastfeeding initiation. The infant barrier was one, which is the inability of the baby to suck breast milk. Some of them indicated that they tried to feed their babies immediately after birth but that they could not suck. Therefore, they had to feed them formula even though formula is not recommended as a replacement for breast milk because of its limitations such as a high risk of contamination and low antibodies, enzymes and hormones [1]. These findings appear consistent with research by Darmstadt et al. [21], Haider et al. [24] and Choudhury et al. [23].

In following these practices, rural women gained knowledge from elder female relatives, who had experiential knowledge. Like Akter et al.’s findings, the advice offered to women by their elders was given with the well-meaning intention to safeguard the well-being of both mother and baby [5]. Therefore, women mostly accepted the advice of elders who were influential in their family and community. A lack of education and strong religious and cultural beliefs prevented women from changing their behavior on following traditional care practices during the postnatal period, as deeply embedded cultural practices change slowly over time [4,5,6,15,16]. 

### Weaknesses and Strengths of the Study

The application of ethnographic research in this study has some obvious limitations. This study was conducted in 2011, about a decade ago, in a select place, and participants were identified purposively from a village different from other rural communities in Bangladesh. Therefore, the generalizability of the findings of this research is limited. A comparison with other areas could increase the comprehensiveness and acceptability of this research. Participants in this research were mothers who had a child under five. Therefore, a large number of individuals who may have had opinions about postnatal care in the village were excluded from this research. A key strength of this study lies in its application of ethnography as a method, for gaining a deep understanding of rural women’s everyday lives and practices during the postnatal period. More particularly, the application of classical ethnography helped to gain insights into cultural and social behaviors and to grasp a deep understanding of the everyday life and practice of women in the village. This study was conducted in an area where childbirth and postnatal care had not previously been researched, which gave me the opportunity to work with people who had not previously been exposed to research.

## 5. Conclusions

This study concludes that the postnatal period is considered significant for maternal and newborn health and is strongly associated with various cultural rituals, beliefs and practices. This study focuses on cultural practices along with education and poverty that play a large part in women’s experiences during childbirth and the postnatal period, which have an effect on the choices of women regarding care and support from a healthcare facility. The majority of women in this study preferred to give birth at home with the assistance of a TBA than in a health facility, but for the overall development of postnatal care regarding safe motherhood, it is important to ensure the presence of an SBA at every birth event, and to strengthen the quality of care in health facilities to achieve the SDGs. While the government is committed to implementing the Safe Motherhood Initiative and trying to achieve the SDGs in regard to maternal and neonatal health, it is not possible to achieve these unless culturally and socially rooted and embedded issues are addressed in policy, planning, programs, research and actions in Bangladesh.

## Data Availability

The original contributions presented in the study are included in the article, further inquiries can be directed to the corresponding author.
